# Burden of Respiratory Syncytial Virus (RSV) on Severe Acute Respiratory Infection and Projected Cost Offsets of Implementing a Regional Adult RSV Vaccination Programme

**DOI:** 10.3390/vaccines14100882

**Published:** 2026-10-05

**Authors:** Liam Townsend, T. J. Bourke, Azonim Egwu, Samuel Zen Kai Yap, Fergal Howley, Brian D. Kent, Conor Woods, Aileen Conway, Brendan Crowley, Colm Kerr, Rose-Anne Kenny, Colm Bergin

**Affiliations:** 1 Department of Infectious Diseases, St James’s Hospital, D08 NHY1 Dublin, Ireland; 2 Department of Clinical Medicine, Trinity College Dublin, D02 R590 Dublin, Ireland; 3 Department of Respiratory Medicine, St James’s Hospital, D08 NHY1 Dublin, Ireland; 4 Department of Endocrinology, St James’s Hospital, D08 NHY1 Dublin, Ireland; 5 Department of Virology, St James’s Hospital, D08 NHY1 Dublin, Ireland; 6 Department of Medical Gerontology, St James’s Hospital, D08 NHY1 Dublin, Ireland

**Keywords:** SARI, RSV, vaccination, cost analysis

## Abstract

**Objective:** RSV is a significant contributor to severe acute respiratory infection (SARI) admissions. Despite national recommendations for RSV vaccination in Ireland, they have not been implemented due to cost concerns and evidence gaps. We assessed RSV-SARI tertiary healthcare engagement to determine healthcare utilisation costs and potential savings in patients following RSV vaccination. **Methods:** RSV SARI admissions over two winter seasons (2024/25 and 2025/26) at a tertiary centre in Ireland were assessed. Sociodemographic and disease-specific variables were recorded, and their relationship with outcomes investigated. Potential cost offsets which may present following a successful RSV vaccination campaign were calculated. **Results:** There were 168 RSV admissions during the study (54% aged ≥75). Median length of stay was 6 days and was significantly longer in patients aged ≥75. Having a chronic neurological disease was associated with increased length of stay. The 90-day readmission rate was 24%. Vaccinating ≥75-year-olds in the hospital catchment area would cost €3,712,500, while predicted admission avoidance post-vaccination would save €1,197,692, or €111.77 per vaccine administered. Vaccine costs of €49 would be cost-neutral at the hospital level. **Conclusions:** RSV disproportionately affects patients ≥75 years of age. Projected vaccine cost offsets in this population are substantially different from those previously used to inform national policy.

## 1. Introduction

Respiratory tract infections, in particular severe acute respiratory infections (SARI), account for a large volume of hospital admissions during winter [1,2]. This places additional strain on hospital services, with downstream consequences for hospital outbreaks, healthcare worker illness, and antimicrobial consumption [3,4,5,6,7]. This hampers overall hospital function and impacts patient access [8,9]. Mitigating the volume and severity of SARI can be achieved through optimising vaccination against recognised pathogens, which may also reduce the economic cost associated with healthcare usage [10]. SARS-CoV-2 and Influenza both have established vaccine programmes, resulting in reductions in hospitalisation and severe illness attributed to these pathogens [11,12]. Respiratory Syncytial Virus (RSV) vaccination in adults has more recently been shown to be safe, with durable efficacy [13,14,15]. The National Immunisation Advisory Committee (NIAC) in Ireland recommends RSV vaccination for all adults aged 75 and older, adults aged 60–74 with underlying health conditions, and adults under the age of 60 in long-term care facilities, with no vaccine recommendations for healthcare workers [16,17]. In April 2026, the Health Information and Quality Authority in Ireland published the findings of its health technology impact assessment and did not find a favourable cost/benefit outcome. As such, it does not recommend reimbursement for the older population at present cost, despite recognising that seasonal RSV surges can lead to scheduled care being delayed and undermine the resilience of the healthcare system [18]. Thus, RSV vaccination in adults is not currently funded nationally in Ireland. The cost/benefit assessment acknowledged that there is an absence of data on RSV disease epidemiology in Ireland; however, as more data becomes available, immunisation strategy decisions may need to be reassessed to ensure that these represent an efficient and equitable use of healthcare resources.

We aimed to assess the burden of RSV-confirmed SARI admissions in adults at a tertiary referral centre across two respiratory viral seasons, and to examine the differences in disease burden and outcomes between patients across the adult age criteria for RSV vaccine recommendations in Ireland. We subsequently performed a simplified economic analysis to assess the potential impact of implementing RSV vaccination guidelines in relevant patient populations within our catchment area. 

## 2. Methods

### 2.1. Clinical Setting and Cohort Characteristics 

This was a combined prospective and retrospective study at St James’s Hospital, Dublin, an adult tertiary referral centre in Ireland. Patients admitted with RSV-confirmed SARI during the preceding 2024/25 respiratory viral season were retrospectively identified. Prospectively, all patients admitted with SARI during the 2025/26 respiratory viral season were followed. Patients with RSV-confirmed SARI underwent enhanced data collection and outcome assessment. SARI was defined in accordance with the National Health Protection Surveillance Centre definition, namely a hospitalised person with acute respiratory infection, with at least one of the following symptoms: cough, fever, shortness of breath, or sudden onset of anosmia, ageusia, or dysgeusia, with onset of symptoms within 14 days prior to hospital admission [19]. 

### 2.2. Data Collection

Data regarding patient demographics, health status, and outcomes from their SARI admission were recoded. Demographic data captured included age, sex, and smoking history, while comorbidities and total number of comedications were also recorded. Specific comorbidities referenced by national immunisation recommendations were noted, namely chronic heart disease, chronic respiratory disease, chronic kidney disease, chronic liver disease, chronic neurological disease, diabetes mellitus, and receipt of immunosuppressive therapy. The conditions included in these definitions are shown in Table S1. Frailty was assessed in patients aged 65 years or older using the Clinical Frailty Scale (CFS). 

Clinical data recorded included admission C-reactive protein (CRP) levels, admission chest X-ray findings, and severity of illness. Severe illness was defined as a composite of patients requiring non-invasive ventilation, intensive care unit (ICU) admission, and death, similar to severity scales used for other SARI causes [20]. Length of stay, intercurrent complications/concurrent diagnoses, need for input from allied health professionals during inpatient stay, and 30- and 90- day readmission rates following discharge were also recorded. 

### 2.3. Ethics Statement

The study was approved by the St James’s Hospital Research and Innovation Committee (project ID 5612, 5 November 2025). Written consent was not required. 

### 2.4. Statistical Analysis

Descriptive statistics are reported as means with standard deviations (SD) and medians with interquartile ranges (IQR), as appropriate. Univariate analysis using Pearson’s Chi-square test, Wilcoxon ranked-sum test, and ANOVA assessed differences in pre-morbid and disease-specific characteristics across the RSV vaccine recommendation age cohorts (<60, 60–74, ≥75 years of age). Univariate linear regression assessed the relationship between length of stay and variables of interest, with subsequent multivariate linear regression used to analyse these variables further. Collinearity in models was assessed by calculating variance inflation factors. 

Economic analysis of vaccine cost offsets and hospital-level cost-neutrality in patients aged ≥75 years utilised currently available list prices from the health technology impact assessment on RSV vaccine cost, cost of hospital stays including readmissions, and the published data on the impact of RSV vaccine on SARI admissions and RSV admissions. Vaccine effectiveness was projected at 92% for reducing direct RSV admission, and 15% for reducing all-cause SARI admissions [13]. Catchment estimate of people aged ≥75 years of age was obtained by aligning the National Ambulance Service catchment area with national census data, identifying 25,000 individuals aged ≥75 within our catchment [21]. Projected vaccine coverage was estimated at 90%, equivalent to national Influenza vaccine uptake in >75 year olds [22]. The current vaccine list price for RSV is €165, while a static administration cost of €25 is applied to adult-based immunisation strategies [18]. The reimbursement fee for RSV admissions is calculated using the National Healthcare Pricing Office for Activity-Based Funding. There is a flat fee paid to hospitals for respiratory infection admissions with a length of stay between 3 and 21 days in Ireland, with a base price of €3650. This base price is multiplied by the inlier relative value (3.564 for major respiratory infection, 1.620 for minor respiratory infection) and weighted by the case distribution. This calculation (Inlier Relative Value × Base Price × Tertiary Centre cost, which is equivalent to a 1.01 multiplier, weighted by cases recorded) gives a reimbursement price per patient of €8876.92 [23]. Length of stay and readmission data were generated from the current study. All analyses were performed using Stata version 19.0 (Stata Statistical Software), while Figures were produced using BioRender.com (https://biorender.com/, accessed on 30 September 2026). 

## 3. Results

### 3.1. SARI Burden, RSV Admissions and Cohort Outcomes

**Figure 1:**
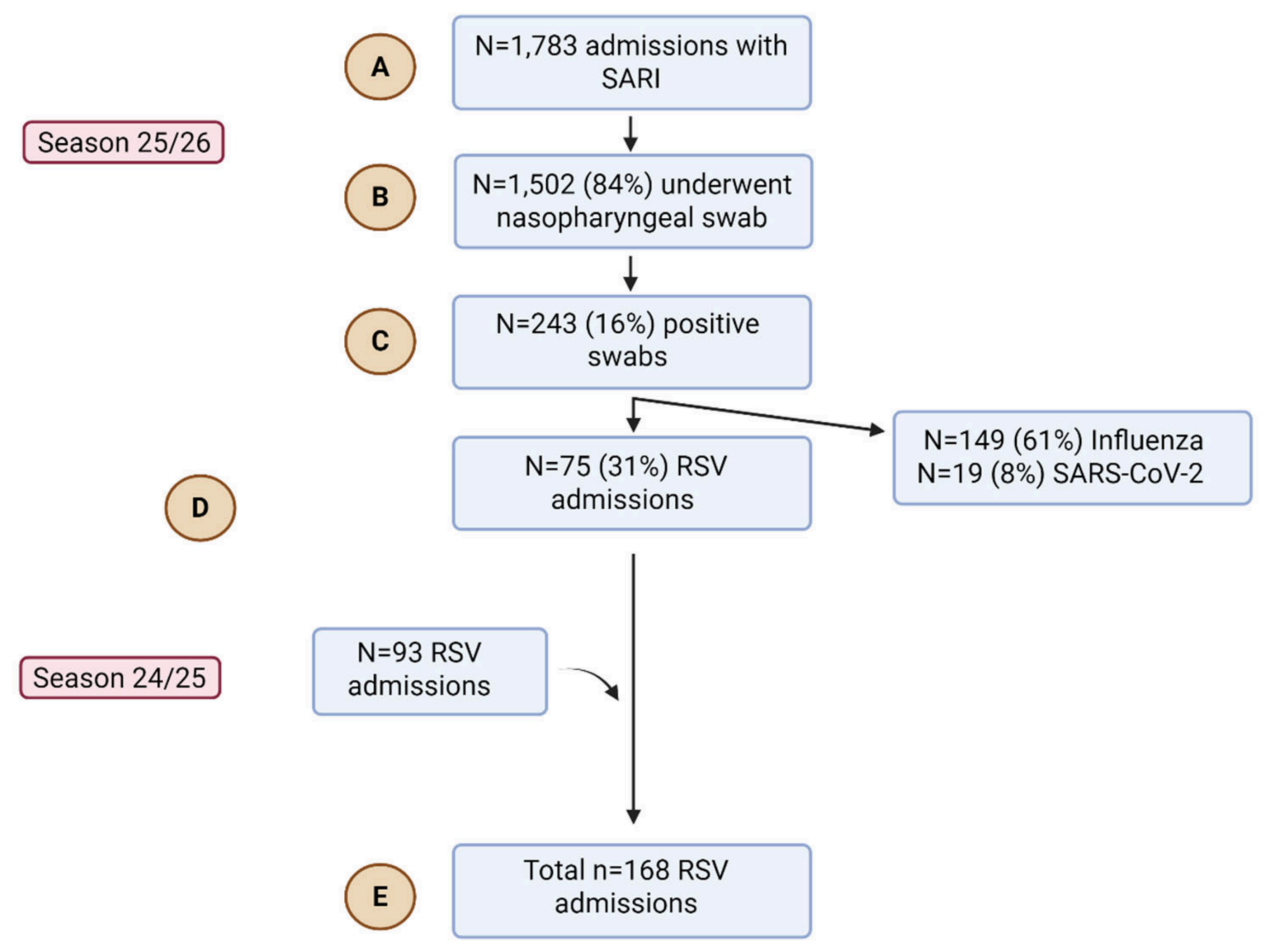
Patient consort diagram. Demonstrating (**A**) SARI admissions during the 2025/26 viral respiratory season, with (**B**) proportion who underwent nasopharyngeal sampling for Influenza, SARS-CoV-2, and RSV PCR, and (**C**) subsequent overall positivity rate. There were (**D**) n = 75 RSV cases, and a further n = 93 identified from the preceding 2024/25 viral respiratory season, resulting in (**E**) n = 168 RSV cases for inclusion in analysis.

There were 1783 SARI admissions via the Emergency Department of St James’s Hospital between December 2025 and April 2026. There were no positive RSV cases admitted that season prior to December 2025. Of those, n = 1502 (84%) underwent nasopharyngeal PCR testing for RSV, Influenza A and SARS-CoV-2. Positive results were found in 16% of swabs (243/1502). The most common pathogen isolated was Influenza A, with n = 75 cases of RSV infection. In the preceding 2024/2025 RSV season, there were n = 93 admissions with PCR-confirmed RSV between December 2024 and April 2025, resulting in a total of n = 168 RSV cases eligible for analysis. Data regarding the total SARI admissions during the 2024/2025 season is not available; as such, the burden of RSV infection amongst the total SARI admissions cannot be estimated for this season. A summary of these results is shown in Figure 1.

A total of 95 (57%) were aged 75 or older, while 39 (23%) were aged between 60 and 74, all of whom had a comorbidity that met RSV vaccination eligibility criteria. There were 34 (20%) admissions aged younger than 60, who were outside of current vaccine recommendations. A total of 22 (13%) patients with RSV were admitted from long-term care facilities, all of whom were older than 60. There were significant differences in pre-morbid characteristics across the age groups. Complete cohort characteristics are shown in Table 1. Those aged ≥75 had a higher number of comorbidities (median 3, IQR 2), as well as a higher burden of chronic neurological disease (21%). Patients aged <60 years had significantly less cardiovascular disease and fewer comedications than those aged 60 or older. 

There were discrete differences in disease outcomes across the groups. The median length of stay in the total cohort was 6 days (IQR 9), with those aged ≥75 having a significantly increased length of stay. There was a significantly increased association with allied health professional input and increasing age. A total of 2216 bed days were used. Of these, 1951 (88%) were occupied by patients who met RSV vaccine recommendations, and 1,583 (71%) were occupied by patients aged ≥75. Patients aged <60 years had fewer additional diagnoses made during their inpatient stay. The most common additional diagnosis was acute kidney injury (21/168, 13%), followed by decompensated heart failure (16/168, 10%). Additional diagnoses recorded are available in Table S2. There were no differences in the incidence of severe disease across the cohorts, but patients aged 60–74 had a significantly higher mortality rate than those aged <60 years of age.

The 90-day readmission rate in the overall cohort was 24%, with no differences between age groups. Of the 146 patients admitted from home rather than a long-term care facility, 15 (10%) required transfer to a convalescence facility prior to returning home. 

### 3.2. Factors Associated with Increased Length of Stay

Given the significant differences in length of stay evident with advancing age, other pre-morbid factors influencing length of stay were assessed. In addition to age, total comorbidity count was associated with increased length of stay. The relevant comorbidities associated with vaccine eligibility were assessed individually, with chronic neurological disease associated with a significantly increased length of stay. In the n = 119 patients aged 65 or older, increased CFS score was also associated with extended length of stay (Table 2). 

These factors were interrogated using two multivariable linear regression models. The first assessed the influence of the significant variables on univariate analysis in the total n = 168 cohort (age category, comorbidity count, and chronic neurological disease), while the second focused on the cohort aged 65 and older, with CFS score included as a variable in this model. Chronic neurological disease remained significantly associated with increased length of stay under both models, while the influence of age cut-offs was no longer significant under either model (Table 3). 

All variables shown were included in the multivariable logistic regression model. Variance inflation factors: age category 1.09, comorbidity count 1.10, chronic neurological condition 1.08, and clinical frailty scale score 1.26.

### 3.3. Economic Assessment of Implementing an RSV Vaccine Programme 

Given the impact of chronic neurological disease on length of stay and the significant age-associated differences in its population distribution, we assessed the economic cost of implementing an RSV vaccination programme in the ≥75-year-olds in our catchment area. There are an estimated 25,000 patients aged ≥75 years of age in our hospital catchment area. With projected vaccine coverage of 90%, this is equivalent to 22,500 total vaccines. Based on the current list price per vaccine of €165, the total drug cost of vaccinating the ≥75 cohort is €3,712,500. 

Cost savings were projected using a hospital reimbursement of €8,876.92 for patients admitted for 3–21 days, known vaccine effectiveness rates, and the length of stay data from our current study. The potential cost mitigated in preventing RSV admissions based on 92% vaccine effectiveness is €318,723. The potential cost savings from non-RSV SARI admissions based on a 15% reduction in all-cause SARI admissions is €805,661. The combined projected costs mitigated from SARI admissions following RSV vaccination in the ≥75-year-olds is €1,124,384. This equates to a total net expenditure of €2,588,116, equivalent to €115.03 per patient vaccinated. 

Given the observed 23% 90-day readmission rate in the RSV-SARI cohort, there is a potential additional cost mitigation of €73,308, resulting in an overall net expenditure of €2,514,808, equivalent to €111.77 saved per patient vaccinated. The complete breakdown of costs is shown in Table 4. For vaccination to be cost-neutral versus predicted acute inpatient savings, vaccine cost would need to be capped at €49.97 (RSV admission and non-RSV SARI admission savings divided by vaccines needed, €1,124,393/22,500), while to be cost-neutral at the hospital level when readmissions are also considered, vaccine cost would need to be capped at €53.23 (RSV admission, non-RSV SARI admission and readmission savings divided by vaccines needed, €1,197,701/22,500).

The impact of variations on the model parameters used was investigated using one-way sensitivity analysis. The variables assessed were changes in vaccine costs, changes in vaccine uptake, changes in vaccine effectiveness, changes in readmission rates, and changes in admission costs. This mirrored the approach taken in the national health technology assessment. Unsurprisingly, the variable with the most influence on per-patient price was overall vaccine cost (range of €30.24–€90.24), while changes in reimbursement costs for hospital stay also showed a large influence on hospital-level cost-neutral vaccine prices (range €38.06–€51.50 per patient). The complete one-way sensitivity analysis is shown in Table S3. 

We did not include logistic costs for vaccine administration in our model. The health technology assessment applied a static administration cost of €25 to adult-based immunisation strategies. In our cohort, this would be equivalent to €562,500 (€25 × 22,500 patients). A total of 100% vaccine coverage would carry a logistical cost of €625,000, while vaccine uptake of 65%, which was the lowest considered in our sensitivity analysis, would have an associated logistical cost of €406,250.

## 4. Discussion

This study demonstrates the burden of illness caused by RSV SARI during the winter season and the potential costs mitigated through a targeted RSV vaccine programme at a single hospital site, in line with national vaccination recommendations. More than half of the patients over the preceding two winter seasons admitted at the study site with RSV-associated SARI were older than 75 years of age. Patients ≥75 have a significantly increased length of stay compared to younger patients, have increased requirement for input from allied health professionals, and are more likely to have a concurrent diagnosis identified during their inpatient stay. Almost one in four patients admitted with RSV are re-admitted within 90 days. The estimated cost of RSV-related SARI admissions is substantial, and our crude cost analysis verifies that this could be reduced by almost 1.2 million euros at a single hospital site with the introduction of vaccination for patients aged ≥75 in our catchment area. 

RSV was identified in 4% of all SARI admissions to our institution, similar to rates reported globally [24,25]. We also report a 7% mortality, in keeping with rates reported in other studies [26,27]. The onset of RSV was slightly later than in previous seasons, with the first patient admitted with RSV being seen in December 2025. This is reflective of national patterns of RSV reporting for the 25/26 season [28].The median length of stay in our cohort is similar to international comparators, and patients aged 75 and over had a greater length of stay [29]. Patients ≥75 years of age accounted for 71% of all bed days used, despite accounting for only 57% of RSV SARI admissions. A pre-existing diagnosis of a chronic neurological disease was also associated with increased length of stay, the most common of which is dementia. Dementia has previously been identified as a better predictor of poor outcome in RSV than age alone [30]. In addition to a prolonged length of stay in the ≥75 cohort, there is greater disease complexity, with an increase in concurrent diagnoses during inpatient stay and an increase in allied health professional input. RSV-associated heart failure and acute kidney injury, which are the most common additional diagnoses seen in our cohort, are associated with poorer long-term outcomes [31,32]. We also observe almost one in four patients readmitted within 90 days of discharge, which is in line with prior data [33]. A total of 10% of patients required off-site convalescence prior to returning home. 

While having a chronic neurologic disease is the strongest predictor of increased length of stay, this is a heterogeneous group of conditions predominantly composed of dementia syndromes and cerebrovascular disease with no established disease registry. This makes vaccination in this specific cohort challenging. However, there is a significantly higher burden of chronic neurological disease in the ≥75 cohort, advocating for an age-based approach rather than one based on a single comorbidity. Furthermore, higher Clinical Frailty Scale scores were associated with increased length of stay on univariate analysis, and frailty is highly correlated with chronological age [34]. 

Given that national vaccine recommendations are for RSV vaccination in the ≥75 group, and there is readily available population-level data for the size of this cohort, an economic analysis of RSV vaccination in this group was performed. Our estimates on cost aversion based on RSV vaccination are similar to the costs quoted in other jurisdictions [35]. The maximum projected vaccine price to be price-neutral for tertiary healthcare engagement ranges from €49.94 to €53.23. This is more than twice the €20 per vaccine cost projected in the Health Technology Impact Assessment for cost-effectiveness [18]. Reasons for this difference may relate to logistical and distribution costs, which were not factored directly into our model. However, the Health Technology Impact Assessment also acknowledged the limitations of their models based on the quantity and quality of available evidence. By utilising clinical data from the most recent viral season, we address some of these limitations. Following one-way sensitivity analysis, we found vaccine cost to be the strongest influence on net cost per patient, with price differences ranging from €30 to 90 per patient. Changes in admission costs were also identified as having a strong influence on price, with vaccine costs ranging from €104 to €120. The switch in Ireland from per diem reimbursement for hospital stay to an activity-based funding mechanism for respiratory infections, whereby a fixed amount is paid to the hospital regardless of length of stay, has not been associated with an increase in efficiency [36]. Any reversion to a per diem reimbursement policy would have a large impact on any economic analysis. It is likely that additional cost savings can be obtained through reduction in emergency department and primary care visits following vaccination, as well as reduced requirements for post-discharge convalescence [37,38]. While this data is a projection, we can look to other jurisdictions where the introduction of RSV vaccination to patients aged 75 and older led to a significant reduction in admissions [39,40]. 

There are several limitations to our study that warrant comment. Given that a portion of this study was retrospective, there may be missing/non-recorded data. Ongoing prospective data collection will help mitigate its impact. As there is no healthcare claim system in Ireland, the attributable cost of hospital stay is based on costs used in national health economic evaluations and cannot be personalised to each individual patient. Costs for major and minor respiratory infections in Ireland are paid at a flat rate for any stay between 3 and 21 days, and are not adjusted based on individual patient outcomes. The seasonal variation associated with RSV may result in significant changes in admission burden and illness severity from year to year, regardless of vaccination. Vaccination rates are not known, with vaccine uptake of 90% used here, using Influenza vaccine uptake as a corollary. The national health technology assessment assumed a vaccine uptake of 88.4% in the ≥75-year-old cohort, similar to our estimates. One-way sensitivity did not show a large cost variation even when vaccine uptake rates were modified. Costs of readmission are based on the assumption that length of stay is similar to the preceding admission. We did not account for the logistical costs of vaccine rollout within our model, but these can be mitigated by utilising pre-existing Influenza and SARS-CoV-2 models. Crude costs using €25 as a base price have been calculated. Potential savings from reduced emergency department and primary care attendances following vaccination were not included, nor were the costs of ongoing convalescent care, which was required in 10% of RSV-SARI patients prior to returning home. The actual cost savings may be much greater than stated here, including mitigating the opportunity cost of cancelled semi-elective admissions due to SARI bed pressures. While the challenges posed by SARI are global, cost analyses will vary across jurisdictions, and our findings may not be directly translatable to other healthcare systems.

## 5. Conclusions

We demonstrate that RSV SARI admissions disproportionately affect patients aged ≥75 years. The threshold for RSV vaccination to be cost-neutral at the hospital level in this study is substantially different from that projected in the initial national vaccine assessment publication and should influence our regional vaccination policy.

## Figures and Tables

**Table 1:** Demographic and disease characteristics stratified by vaccine eligibility.

	Total Cohort n = 168	Age <60 n = 34	Age 60–74 n = 39	Age ≥75 n = 95	
Sex, female; n (%)	98 (58)	20 (59)	24 (62)	54 (57)	Χ^2^ = 0.26, *p* = 0.88
Comorbidity count; median (IQR)	2 (2)	2 (1)	3 (2)	3 (2)	r^2^ = 0.10, *p* = 0.0002
Comorbidities; n (%) - Type 2 diabetes mellitus - Active malignancy - Chronic neurological disease - Chronic kidney disease - Cardiovascular disease - Chronic lung disease	29 (17) 38 (23) 23 (14) 13 (8) 49 (29) 74 (44)	2 (6) 8 (24) 0 (0) 0 (0) 3 (9) 15 (44)	5 (13) 12 (31) 1 (3) 2 (5) 13 (33) 24 (62)	20 (21) 18 (19) 20 (21) 11 (12) 33 (35) 35 (37)	Χ^2^ = 6.15, *p* = 0.05 Χ^2^ = 2.23, *p* = 0.33 Χ^2^ = 10.94, *p* = 0.004 Χ^2^ = 5.19, *p* = 0.08 Χ^2^ = 8.57, *p* = 0.01 Χ^2^ = 6.84, *p* = 0.03
Comedication count; median (IQR)	8 (5)	5 (4)	9 (4)	9 (5)	r^2^ = 0.20, *p* < 0.0001
Iatrogenic immunosuppression; n (%)	24 (14)	4 (12)	7 (18)	13 (14)	Χ^2^ = 0.63, *p* = 0.73
Current smoker, yes; n (%)	29 (17)	10 (29)	8 (21)	11 (12)	Χ^2^ = 11.18, *p* = 0.08
Length of stay, days; median (IQR)	6 (9)	6 (6)	6 (6)	7 (12)	r^2^ = 0.05, *p* = 0.01
Complication/concurrent issue	73 (53)	8 (24)	20 (57)	45 (47)	Χ^2^ = 7.30, *p* = 0.03
Admission CXR consistent with infection; n (%)	79 (47)	12 (35)	26 (74)	41 (43)	Χ^2^ = 8.49, *p* = 0.01
Admission CRP, mg/L; median (IQR)	49 (84)	51 (70)	53 (106)	43 (80)	r^2^ = 0.01, *p* = 0.55
Allied health * input required, yes; n (%)	117 (70)	17 (50)	26 (67)	74 (78)	Χ^2^ = 9.43, *p* = 0.009
Severe disease; n (%)	26 (15)	4 (12)	9 (23)	13 (14)	Χ^2^ = 2.31, *p* = 0.31
Death; n (%)	11 (7)	0 (0)	6 (15)	5 (5)	Χ^2^ = 7.61, *p* = 0.02
Readmission at 30 days; n (%)	26 (15)	7 (21)	6 (15)	13 (14)	Χ^2^ = 0.87, *p* = 0.65
Readmission at 90 days; n (%)	41 (24)	11 (32)	8 (21)	22 (23)	Χ^2^ = 2.29, *p* = 0.32

* Allied health: Any of physiotherapy, occupational therapy, social work, speech and language therapy, and clinical nutrition. CXR: chest X-ray, CRP: C-reactive protein. Differences were assessed using ANOVA (r^2^) and Pearson’s Chi-square test (Χ^2^). Comorbidities and iatrogenic immunosuppression are defined in Supplemental Table S1. Severe disease is defined as a composite of patients requiring non-invasive ventilation, intensive care unit (ICU) admission, and death.

**Table 2:** Factors associated with increased length of stay.

	Statistic
Age	r^2^ = 0.21, *p* = 0.005
Sex, female	r^2^ = 0.02, *p* = 0.76
Current smoker	r^2^ = 0.01, *p* = 0.95
Clinical frailty scale score	r^2^ = 0.22, *p* = 0.02
Comorbidity count - Diabetes - Active malignancy - Chronic neurological disease - Chronic kidney disease - Cardiovascular disease - Chronic lung disease	r^2^ = 0.17, *p* = 0.03 r^2^ = −0.11, *p* = 0.17 r^2^ = −0.02, *p* = 0.82 r^2^ = 0.31, p<0.0001 r^2^ = 0.03, *p* = 0.69 r^2^ = 0.02, *p* = 0.77 r^2^ = −0.02 p = 0.80
Comedication count	r^2^ = −0.11, *p* = 0.17

**Table 3:** Multivariable analysis of factors associated with increased length of stay.

	Total Cohort (n = 168)		≥65 Years of Age (n = 119)	
	β coefficient (95% CI)	*p* value	β coefficient (95% CI)	*p* value
Age <60 60–74 ≥75	Reference −0.64 (−9.96–8.68) 4.98 (−3.43–13.39)	N/A 0.89 0.24	N/A Reference 3.19 (−7.36–13.73)	N/A 0.55
Comorbidity count	1.23 (−0.35–2.81)	0.13	1.04 (−1.05–3.14)	0.33
Chronic neurological disease	16.57 (7.53–25.60)	<0.0001	14.82 (3.85–25.79)	0.009
Clinical frailty scale score	N/A	N/A	0.90 (−12.27–14.07)	0.13

**Table 4:** Cost analysis of RSV vaccination of patients ≥75 years of age.

**Vaccine Costs**		
Individuals aged ≥75 in catchment area	25,000	
Vaccines needed, based on 90% vaccine uptake	25,000 × 0.90 = 22,500	
Cost per vaccine, based on list price	€165	
Vaccine cost in ≥75 cohort	22,500 × €165	€3,712,500
**Projected impact of vaccination on RSV admissions**		
RSV admissions 25/26 − Aged ≥75 − Projected 90% vaccine uptake	76 − 43 − 43 × 0.9 = 39	
Projected hospitalisations prevented ≥75 years with vaccination	39 × 0.92 = 36	
Median admission length in aged ≥75	7 days	
Average cost per admission	€8853.42	
Savings for RSV admissions	36 × 8,853.42	€318,723
**Projected impact of vaccination on non-RSV SARI admission costs**		
SARI admissions 25/26 − Non-RSV − Non-RSV ≥75 years old − Assuming 90% vaccine uptake	1783 1693 671 671 × 0.90 = 604	
Projected non-RSV SARI admissions prevented in those aged ≥75 with vaccination	604 × 0.15 = 91	
Median non-RSV SARI admission length in those aged ≥75	8 days	
Average cost per admission	€8853.42	
Savings for non-RSV SARI admissions	91 × 8853.42	€805,661
**Projected vaccine impact on RSV and non-RSV SARI admission costs**		
Direct RSV cost savings + non-RSV SARI cost savings	€318,723.12 + €805,661.22 = €1,124,384	
Net cost (vaccine cost–cost savings)	€3,712,500–€1,124,384.34	€2,588,116
Net cost per patient aged ≥75	€2,588,116/22,500	€115.03
**Projected re-admission cost savings**		
90-day readmission rate	23%	
Cost of readmission (RSV admission cost × readmission rate)	€318,732 × 0.23	€73,308
Total projected savings (RSV + non-RSV SARI + post RSV-readmission costs)	€318,723 + €805,661 + €73,308	€1,197,692
Net cost (vaccine cost–total projected savings cost)	€3,712,500–€1,197,692	€2,514,808
Net cost per patient aged ≥75	€2,514,808/22,500	€111.77

## Data Availability

The original contributions presented in this study are included in the article/Supplementary Material. Further inquiries can be directed to the corresponding author.

## Supplementary Materials

The following supporting information can be downloaded at: https://www.mdpi.com/article/10.3390/vaccines14100882/s1, Table S1: List of chronic conditions by system; Table S2: Frequency of concurrent diagnoses/intercurrent complications in vaccine-preventable SARI admissions; Table S3: One-way sensitivity analysis of economic model variables.
