# Supplementary material for: Burden of Respiratory Syncytial Virus (RSV) on Severe Acute Respiratory Infection and Projected Cost Offsets of Implementing a Regional Adult RSV Vaccination Programme
Source: Vaccines (Basel). 2026 Oct 5;14(10):882. doi: 10.3390/vaccines14100882 (PMC13709742; doi:10.3390/vaccines14100882)
Supplement: Supplementary file 1 [file vaccines-14-00882-s001.zip › vaccines-4547160-supplementary.pdf]

**Table S1.** List of chronic conditions by system.

| | |
|---|---|
| Active malignancy | Solid tumour<br>Haematological malignancies |
| Chronic neurological disorder | Alzheimer's dementia<br>Vascular Dementia<br>Dementia with Lewy Body<br>Unspecified dementia<br>Cognitive impairment<br>Motor Neuron Disease<br>Multiple Sclerosis<br>Parkinson's disease<br>Cerebral infarction<br>Cerebral haemorrhage |
| Chronic kidney disease | Stage 3 chronic kidney disease<br>Stage 4 chronic kidney disease<br>Stage 5 chronic kidney disease<br>Receiving renal replacement therapy |
| Cardiovascular disease | Heart failure with reduced ejection fraction<br>Heart failure with preserved ejection fraction<br>Ischaemic heart disease<br>Myocardial infarction<br>History of coronary artery bypass grafting<br>Ischaemic cardiomyopathy<br>Atrial fibrillation |
| Chronic lung disease | Chronic obstructive pulmonary disease<br>Asthma<br>Asthma/COPD Overlap Syndrome<br>Pulmonary fibrosis, all-cause |
| Immunosuppressive therapy | Haematopoietic stem cell transplant<br>Solid organ transplant recipients<br>Corticosteroid equivalent to ≥20mg prednisolone daily<br>Anti-CD20 therapy<br>Anti-CD52 therapy<br>Anti-TNF therapy<br>Cytotoxic chemotherapy<br>JAK inhibitors<br>IL-6 inhibitors<br>Disease-modifying antirheumatic drug therapy |

**Table S2.** Frequency of concurrent diagnoses/intercurrent complications in vaccine-preventable SARI admissions.

| Complication | Frequency n (%) |
|---|---|
| Acute kidney injury | 21 (13) |
| Acute decompensated heart failure | 16 (10) |
| Concurrent bacterial pneumonia | 12 (7) |
| New or decompensated atrial fibrillation | 10 (6) |
| Delirium | 9 (5) |

Total cohort n=168. Only complications occurring in ≥ 2% of patients shown.

**Table S3.** One-way sensitivity analysis of economic model variables.

| **(A) Changes in vaccine cost** | | | | |
|---|---|---|---|---|
| Vaccine price | Vaccine cost | Net cost per patient (No readmissions) | Net cost per patient (With readmissions) | |
| €80 | €1,800,000 | €30.24 | €27.00 | |
| €100 | €2,250,000 | €50.24 | €47.00 | |
| €120 | €2,700,000 | €70.24 | €67.00 | |
| €140 | €3,150,000 | €90.24 | €97.00 | |
| **(B) Changes in vaccine uptake** | | | | |
| Vaccine uptake (%) | Number immunised | Hospital-level cost-neutral vaccine price (No readmissions) | Hospital-level cost-neutral vaccine price (With readmissions) | |
| 95 | 23,750 | €47.27 | €53.00 | |

| | | | | |
|---|---|---|---|---|
| 85 | 21,250 | €42.29 | €53.00 | |
| 75 | 18,750 | €37.32 | €53.00 | |
| 65 | 16,250 | €32.34 | €53.00 | |
| **(C) Changes in vaccine effectiveness** | | | | |
| Vaccine Effectiveness (%) | Net cost per patient (No readmissions) | Net cost per patient (With readmissions) | Hospital-level cost-neutral vaccine price (No readmissions) | Hospital-level cost-neutral vaccine price (With readmissions) |
| 0.95 | €114.78 | €111.43 | €45.20 | €53.57 |
| 0.89 | €115.70 | €112.56 | €44.37 | €52.44 |
| 0.86 | €116.16 | €113.13 | €43.95 | €51.87 |
| 0.83 | €116.62 | €113.69 | €43.54 | €51.31 |
| **(D) Changes in readmission rates** | | | | |

| Readmission rate (%) | Cost of readmissions | Net cost per patient (With readmissions) | Hospital-level cost neutral vaccine price (With readmissions) | |
|---|---|---|---|---|
| 29 | €1,211,643.28 | €111.15 | €53.85 | |
| 26 | €1,202,116.39 | €111.57 | €53.43 | |
| 20 | €1,183,062.61 | €112.42 | €52.58 | |
| 17 | €1,173,535.72 | €112.84 | €52.16 | |
| 14 | €1,164,008.83 | €113.27 | €51.73 | |
| **(E) Changes in admissions reimbursement** | | | | |
| | Net cost per patient (No readmissions) | Net cost per patient (With readmissions) | Hospital-level cost-neutral vaccine price (No readmissions) | Hospital-level cost-neutral vaccine price (With readmissions) |
| €10,181.43 (15% increase) | €107.78 | €104.05 | €51.50 | €60.95 |

| | | | | |
|---|---|---|---|---|
| €9,738.76 (10% increase) | €110.27 | €106.70 | €49.26 | €58.30 |
| €8,410.75 (5% decrease) | €117.73 | €114.65 | €42.54 | €50.35 |
| €7,968.08 (10% decrease) | €120.22 | €117.30 | €40.30 | €47.70 |
| €7,525.41 (15% decrease) | €122.71 | €119.95 | €38.06 | €45.05 |

Changes across five variables assessed: **(A)** wholesale vaccine price (similar to HIQA HTA assessment), **(B)** changes in vaccine uptake (ranging from 65% - 95%). **(C)** changes in vaccine effectiveness for preventing hospitalisation (ranging from 83% - 95%), **(D)** Changes in readmission rates (ranging from 14 – 29%), and **(E)** Changes in admission reimbursement (+/- 15% from current level). The other variables in each model were maintained constant at levels used in main economic analysis.
